# Burkitt's lymphoma in a young boy progressing to systemic lupus erythematosus during follow-up: a case report and literature review

**DOI:** 10.3389/fped.2024.1348342

**Published:** 2024-03-01

**Authors:** Chenxi Liu, Ci Pan, Yingying Jin, Hua Huang, Fei Ding, Xuemei Xu, Shengfang Bao, Xiqiong Han, Yanliang Jin

**Affiliations:** ^1^ Department of Rheumatology and Immunology, Shanghai Children’s Medical Center, Shanghai Jiao Tong University School of Medicine, Shanghai, China; ^2^ Department of Hematology and Oncology, Shanghai Children’s Medical Center, Shanghai Jiao Tong University School of Medicine, Shanghai, China

**Keywords:** Burkitt’s lymphoma, systemic lupus erythematosus, autologous stem cell transplantation, children, case report

## Abstract

**Introduction:**

Patients with systemic lupus erythematosus (SLE) are at a higher risk of developing cancer, particularly hematological malignancies such as lymphoma and leukemia. However, existing studies on this topic that assess cancer incidence following SLE diagnosis are limited. In addition, SLE can be diagnosed after cancer, although such cases in children have been rarely reported.

**Case report:**

We present the case of a 2.6-year-old boy who presented to our institute with fever and abdominal pain. His physical examination revealed a periumbilical mass, which was pathologically diagnosed as Burkitt's lymphoma. Autologous stem cell transplantation was performed to consolidate the effect of chemotherapy and reduce the risk of cancer relapse. He was diagnosed with SLE 5 years later, following the presentation of a fever with rash, positive autoantibodies, decreased complement, and kidney involvement. At the final follow-up, the patient was still alive and showed no recurrence of Burkitt's lymphoma or disease activity of SLE.

**Conclusion:**

Despite the low frequency of SLE in children with lymphoma, cancer and SLE may be induced by a common mechanism involving B-cell cloning and proliferation. Therefore, hematologists and rheumatologists should be aware of the occurrence of these two conditions during patient follow-up.

## Introduction

Over the past decade, several large-scale studies have elucidated the risk of systemic lupus erythematosus (SLE) in adults. A growing body of evidence has suggested that patients with SLE are at a higher risk of specific malignancies, such as non-Hodgkin's lymphoma (NHL) and leukemia, in comparison with the general population. This may be related to clonal proliferation and high B-cell activation. It has been reported that in SLE populations, the risk of hematological malignancy increased by approximately threefold, whereas endometrial, breast, ovarian, and prostate cancers showed a lower incidence (1, 2). However, it is inappropriate to extrapolate the characteristics of adult-onset SLE to pediatric populations due to the long disease course and the large clinical differences in disease activity, treatment, and organ involvement that exist between pediatric and adult-onset SLE. Moreover, the relationship between lymphoma and autoimmune diseases appears to be bidirectional, although most studies focused on the incidence of lymphoma following autoimmune disease diagnosis, especially in adult cohorts. To date, there is only one case reported on new-onset SLE in an adult woman with a history of lymphoma (3). Here, we report the first case report of a male child diagnosed with Burkitt's lymphoma that subsequently developed into SLE.

## Case presentation

In April 2015, a 2.6-year-old boy was admitted to the Shanghai Children's Medical Center due to persistent fever and abdominal pain that lasted more than 2 months. His physical examination revealed hard lumps near the umbilicus in the left middle and right lower abdomen, with poor motion and no obvious tenderness. A contrast-enhanced abdominal computed tomography (CT) indicated a huge space occupation (6.5 cm × 8.8 cm × 12.4 cm) in the left abdomen and suspected a malignant abdominal tumor. Retroperitoneal partial tumor resection was performed, and the postoperative pathology was consistent with Burkitt’s lymphoma. The contrast-enhanced CT of other organs and bone scans did not show an abnormal space occupation, and bone marrow aspiration did not suggest bone marrow infiltration. Laboratory investigations showed normal white blood cell count, hemoglobin level, and platelet count and a slightly elevated C-reactive protein level (15 mg/L, normal <8 mg/L). Fluorescence *in situ* hybridization was negative for c-Myc, BCL2, and ALK rearrangements. Immune typing was negative for PAS, PASD, CDPP, CK, CGA, SYN, S100, CD3, CD4, ACK1, PGM1, MAC387, CD30, EMA, and PES and positive for CD20, CD79a, Ki67, PAX5, VIM, INI-1, and CD34. The etiological test was positive for Epstein–Barr virus (EBV) and immunoglobulin M (IgM), despite normal DNA quantification of EBV. No autoantibodies were detected at that time, and no significant travel, medical, or family ailment histories were noted. According to the World Health Organization 2016 classification criteria for hematopoietic and lymphoid tumors and St. Jude/Murphy staging criteria (4, 5), the patient was eventually diagnosed with stage 3 Burkitt's lymphoma due to a local but unresectable abdominal mass. Subsequently, he was enrolled in the R4 group of the CCCG-BNHL-2015 chemotherapy regimen since his lactate dehydrogenase level is >4 times the normal value (3532 U/L) (6). The patient underwent radical excision of the abdominal tumor after the fourth course of chemotherapy and completed six courses of chemotherapy. To consolidate the effects of chemotherapy and reduce cancer relapse, the patient underwent an autologous stem cell transplantation (ASCT) at the end of his chemotherapy. The patient was followed up regularly for nearly 4.5 years after ASCT, showing event-free survival.

In August 2020, the patient was presented to our hospital again due to recurrent fever accompanied by a rash that lasted for 2 months. The rash was initially confined to the cheeks and appeared with erythematous scales. It was accompanied by pruritus with partial maculopapular scabs, which gradually spread to the extremities. Physical examination revealed oral ulcers and swollen cervical lymph nodes. The patient denied any other discomfort, such as joint pain, hair loss, or fatigue. etiological examination was negative for serologic EBV, cytomegalovirus, and tuberculosis. Routine blood examination suggested leukopenia (2.97 × 10^9^/L), anemia (98 g/L) with a positive Coombs test, and thrombocytopenia (104 × 10^9^/L). No naive lymphocytes were identified on bone marrow aspiration, and the bone marrow biopsy did not indicate lymphoma relapse. Auto-immunological examination indicated positive antinuclear antibody (ANA) with a titer of 1:1,000. Anti-ribosomal antibody (anti-rRNP), anti-Smith antibody (anti-Sm), anti-SSA/Ro52 antibody (anti-SSA/Ro52), anti-SSA/Ro60 antibody (anti-SSA/Ro60), anti-SSB antibody (anti-SSB), anti-double-stranded DNA antibody (anti-dsDNA), anti-nucleosome antibody, anti-histone antibody, and anti-ribosomal antibodies were all positive, whereas antiphospholipid antibodies were negative. Meanwhile, the level of complement 3 (C3 0.29 g/L, normal at 0.9–1.8 g/L), complement 4 (C4 0.04 g/L, normal at 0.1–0.4 g/L), and the total complement activities (CH50 U/ml, normal at 23–46 U/ml) were also low. Immunoglobulins G and globulins were elevated, with values of 26 g/L (normal at 6.3–15.0 g/L) and 40.8 g/L (normal at 18–32 g/L), respectively, while the rheumatoid factor was normal. Other biochemical tests indicated an elevated erythrocyte sedimentation rate (ESR) level (89 mm/h, normal at 0–20 mm/h) and normal liver and kidney functions. The maximum 24-h urinary protein level was 440 mg, with renal puncture indicating that the pathology coincided with lupus nephritis III + V. No pleural or pericardial effusion was identified on high-resolution CT of the chest or cardiac ultrasound. Ultrasonography of the thyroid and two salivary glands indicated an uneven echo of the thyroid and submandibular glands. The patient denied a family history of connective tissue disease, and whole-exome sequencing revealed no abnormalities. SLE was diagnosed according to the European League Against Rheumatism and the American College of Rheumatology (EULAR/ACR) 2019 diagnostic criteria (7), with a score of 20 (fever, subacute cutaneous lupus, anemia with positive Coombs test, low C3 and C4, and positive anti-Sm and anti-dsDNA). The patient may also have been diagnosed with SLE with features of Sjogren's syndrome since was positive for anti-SSA and anti-SSB and had a fever, high IgG level, leukopenia, elevated ESR level, and even abnormal submaxillary gland echo on ultrasonography.

The patient was administered glucocorticoids, hydroxychloroquine, and mycophenolate. Glucocorticoid administration was discontinued by August 2022 gradually. During the follow-up period, the anticardiolipin antibody began to be positive, and involvement of neuropsychiatric lupus and interstitial lung disease in other organs did not occur. During the most recent follow-up in May 2023, the patient is currently alive and has not experienced a relapse of Burkitt's lymphoma and disease activity of SLE. The latest ANA test was also positive, with a titer of 1:320, and only anti-SSA/Ro52, anti-SSA/Ro60, and anti-rRNP remained positive.

## Discussion

At present, there is a growing body of research focusing on the relationship between autoimmune disease and cancer. Most related data have shown that malignancies are detected only after the diagnosis and treatment of autoimmune diseases, particularly SLE and Sjögren's syndrome. Neoplasia can occur either early or simultaneously. Studies have demonstrated an increased risk of malignancies in SLE patients, especially hematological malignancies and particularly NHL and acute lymphoblastic leukemia (ALL), which appear to be more common than other malignancies (1). The standardized incidence ratio (SIR) of NHL ranged from 4.39 to 7.4 in adult cohorts (2, 8, 9, 10, 11). One study based on the largest number of pediatric-onset SLE patients focused on cancer incidence and found that out of the 1,168 patients, 14 patients developed cancer, with an SIR of 4.13, and 3 patients developed NHL, with an SIR of 4.68 (12). In addition, only a small number of cases of cancer occurrence in children with SLE have been reported. However, it is somewhat reassuring that the incidence in absolute terms still translates into relatively few events. The characteristics of hematological malignancies in children with SLE are summarized in Tables 1 and 2 (12–20). The analysis of this data revealed no strong correlation between SLE severity and tumor development. Among these patients, only three developed into SLE following a diagnosis of ALL (17–19), and one patient underwent ASCT (18). Our case is the first report of a male patient who did not have a genetic predisposition or family history of connective tissue disease that developed into SLE, even secondary Sjögren's syndrome, following diagnosis of Burkitt's lymphoma and ASCT, suggesting a link between cancers and autoimmune disease.

**Table 1:** General information for children with concurrent hematological malignancies and systemic lupus erythematosus (SLE).

Study	Sex	Age of SLE (years)	Race	Hematological malignancy	Time
Merino et al. (12)	Male	9	Hispanic	NHL	Lymphoma occurred 9 years after the diagnosis of SLE
Gokce et al. (13)	Female	6	Turkish	ALL	ALL occurred 10 months after the diagnosis of SLE
Maheshwari et al. (14)	Male	3	Indian	ALL	ALL occurred coexistent with SLE
Saulsbury et al. (15)	Female	7	American	ALL	ALL occurred coexistent with SLE
	Female	12	American	ALL	ALL occurred coexistent with SLE
Bernatsky et al. (16)	Unknown	Unknown	American	NHL	One of the malignancies occurred in the first year after the diagnosis of SLE. The condition of the other two children is unknown.
Sheehan (17)	Female	15	British	ALL	ALL occurred 12 years before the diagnosis of SLE
Steinbach et al. (18)	Male	8.5	Hispanic	ALL	ALL occurred 3.5 years before the diagnosis of SLE; an autologous bone marrow transplant was also completed due to ALL
Al Mayouf et al. (19)	Female	12	Saudi Arabian	ALL	ALL occurred 9 years before the diagnosis of SLE
Jerónimo et al. (20) Our study	Female Male	17 7	Portuguese Chinese	HL NHL	HL occurred coexistent with SLE NHL occurred 5 years before the diagnosis of SLE

NHL, non-Hodgkin's lymphoma; ALL, acute lymphoblastic leukemia; HL, Hodgkin's lymphoma.

**Table 2:** Clinical characters for children with concurrent hematological malignancies and systemic lupus erythematosus (SLE).

Study	Clinical features of SLE	Clinical features of cancer	Treatment for cancer	Treatment for SLE	Prognosis
Merino et al. (12)	Vasculitis, butterfly rash, nephritis, arthritis, ANA+, anti-dsDNA+, C3¯, C4¯, lupus anticoagulant positive, nephritis	Cervical lymphadenopathy	AVBD protocol, including Adriamycin, vinblastine, bleomycin, and dacarbazine, followed by radiotherapy	Steroids, hydroxychloroquine, cyclophosphamide, azathioprine	Alive and relapse-free
Gokce et al. (13)	Abnormal liver function, seizure, leukopenia, Coombs test positive, anemia, thrombocytopenia, C3¯, *ANA*+, anti-dsDNA+, nephritis	Leukopenia, anemia, thrombocytopenia, hepatomegaly	Modified St Jude TXV chemotherapy	Pulse methylprednisolone and azathioprine. HLH-2004 protocol was given due to macrophage activation syndrome	Died because of multiorgan failure and pulmonary hemorrhage
Maheshwari et al. (14)	Fever, arthritis, ANA+, anti-dsDNA+	Hepatomegaly, splenomegaly, pancytopenia	Unknown	Unknown	Unknown
Saulsbury et al. (15)	Fever, seizure, arthritis, ANA 1:960, anti-dsDNA+	Leukopenia	Introduction therapy with vincristine and prednisone.	Unknown	Both clinical symptoms and antibody titers were better than those at diagnosis of SLE in the first 6 months.
	Malaise, weight loss, arthritis, fever, ESR­, ANA 1:960, positive Coombs test, thrombocytopenia	Leukopenia	Introduction therapy with vincristine and prednisone followed by L-asparaginase, cyclophosphamide	Unknown	Remission except for mild thrombocytopenia still persisted during the maintenance chemotherapy
Bernatsky et al. (16)	Unknown	Unknown	Unknown	Unknown	Unknown
Sheehan (17)	Rash, arthritis, Raynaud's phenomenon, leukopenia, ANA > 1:320, ESR­, anti-Sm+	Pancytopenia, lymphadenopathy, hepatosplenomegaly	Treated with UKALL V chemotherapy protocol, including prednisolone, vincristine, methotrexate, and mercaptopurine	Unknown	Unknown
Steinbach et al. (18)	Arthritis, malar rash, fever, weight loss, nephritis, anemia, thrombocytopenia, ESR↑, ANA 1:640, anti-dsDNA+, anti-Sm+, anti-rRNP+, C3↓, C4↓	Fever, pallor, leukocytosis, anemia, thrombocytopenia	Treated with high-risk chemotherapy protocol; underwent autologous bone marrow transplant because of relapse in the period of maintenance therapy	Corticosteroids and intravenous cyclophosphamide	No disease activity after 3 months of treatment
Al Mayouf et al. (19)	Rash, fever, arthritis, pulmonary hemorrhage, seizure, nephritis, ANA 1:2,560, anti-dsDNA+, anti-SSA+, anti-SSB+, C3↓, C4↓, anticardiolipin antibody+	Fever, lymphadenopathy, hepatomegaly, splenomegaly, and pancytopenia	CCG1891chemotherapy protocol, including prednisone, L-asparaginase, vincristine, methotrexate, 6-mercaptopurine, and cyclophosphamide	Prednisone, hydroxychloroquine, and cyclosporine	Unknown
Jerónimo et al. (20) Our study	Arthritis, anemia, leukopenia, nephritis, ANA+, anti-dsDNA+ Rash, oral ulcers, anemia, leukopenia, thrombocytopenia, ANA 1:1,000, anti-Sm+, anti-dsDNA+, anti-SSA+, anti-SSB+, C3↓, C4¯, nephritis	Fever, night sweats, weight loss Fever, abdominal pain	Chemotherapy was not administered due to the complications of SLE. CCCG-BNHL-2015 chemotherapy regimen, and ASCT	Glucocorticoids, cyclophosphamide, and mycophenolate Glucocorticoids, hydroxychloroquine, and mycophenolate	Both the SLE and HL were partially controlled Alive and relapse-free

C3, complement 3; C4, complement 4; anti-dsDNA, the anti-double-stranded DNA antibody; anti-Sm, the anti-Smith antibody; ANA, positive antinuclear antibody; ESR, erythrocyte sedimentation rate; NHL, non-Hodgkin's lymphoma; ALL, acute lymphoblastic leukemia; HL, Hodgkin's lymphoma; ASCT, autologous stem cell transplantation.

Sometimes, clinical manifestations, including arthritis, fever, lymphadenopathy, hepatosplenomegaly, leukopenia, anemia, and thrombocytopenia, are not specific to hematological malignancies or SLE. Therefore, for children with hematological malignancies, autoantibody tests must be completed in time if new symptoms cannot be explained entirely by the tumor or point to rheumatic diseases, and vice versa; bone marrow aspiration, or even bone or lymph node biopsies, should be performed in time if children with SLE have refractory leukopenia, anemia, thrombocytopenia, high lactate dehydrogenase (21), or persistent fever during treatment. Persistent dysplasia/cytopenia, a complicated disease course, multisystem involvement, drugs, and early age at SLE onset may all constitute the development of hematological malignancies (13). However, it is worth noting that ANA is not a specific screening indicator for rheumatic disease. Positive ANA may be found in patients with connective tissue diseases, infectious diseases, and nonspecific musculoskeletal pain (22, 23) and even in healthy populations (24, 25). Therefore, to make a comprehensive judgment, it is necessary to combine other antibodies, such as anti-dsDNA and anti-Smith, and to conduct a long-term follow-up of the immunological indicators of tumor patients with ANA positivity.

Moreover, when lymphoma and SLE are diagnosed in a young patient, differential diagnoses including monogenic lupus and birth immune error, especially autoimmune lymphoproliferative syndrome (ALPS), should be considered. ALPS is relegated to those disorders with variants in the activation-induced cell death pathway mediated by FAS, with somatic or germline variants in FAS, FASL, or CASP11 (26). However, genetic abnormalities alone cannot be used to diagnose ALPS. Usually, chronic (disease course of >6 months) lymphadenopathy and splenomegaly and elevated CD3 ^+ ^TCR*αβ*^+^CD4^−^CD8^−^ cells > 1% total lymphocytes or >2.5% CD3^+^ lymphocytes on flow cytometry are necessary for diagnosis. Other clinical criteria, such as autoimmune cytopenia and elevated vitamin B12, interleukin-10, and interleukin-18 levels may support the diagnosis. Meanwhile, patients with ALPS may also experience autoimmune cytopenia, including Coombs-positive autoimmune hemolytic anemia, immune thrombocytopenia, and autoimmune neutropenia (27). Our patient did not test positive for any gene variants related to ALPS or ALPS-like conditions after repeated analysis of the whole exon sequencing results. This negative result failed to indicate a common genetic background for the co-occurrence of lymphoma and SLE.

In addition, although a meta-analysis showed that mesenchymal stem cell transplantation has a favorable safety profile and good efficacy in improving the activity of lupus nephritis and renal function of SLE patients (28), earlier studies have found that hematopoietic stem cell transplantation (HSCT) or unexpected full hematopoietic engraftment following solid organ transplants may cause autoimmune disorders in both patients and mouse models (29, 30, 31, 32). SLE is a typical autoimmune disease characterized by the presence of many pathogenic autoantibodies (such as ANA, anti-dsDNA, anticardiolipin antibodies, or antibodies against various blood cell components). These autoantibodies may be the product of long-lived plasma cells in the bone marrow and may be present at low titers prior to HSCT without associated pathogenicity. If the regulatory cells that control these self-reactive plasma cells are inhibited by conditioning, the plasma cells can survive the intense lymphatic depletion during HSCT (33). In addition, the re-emergence of autoreactive B or T cells following HSCT may contribute to the autoimmunity in patients. In our case, SLE was diagnosed 4.5 years after ASCT; however, whether SLE-related antibodies were produced before the symptoms appeared, even at the time of development of Burkitt's lymphoma, or after ASCT remains unclear. This reminds us that it is necessary to pay attention to the screening and monitoring of autoantibodies during the process of diagnosis and follow-up.

Similar to other autoimmune diseases, the association between immune disorders, persistent chronic inflammation, and immunosuppressive therapy provides an appropriate background for the relationship between SLE and hematological malignancies. Several studies have suggested that SLE patients had increased viral load, EBV mRNA expression, EBV-directed antibodies, and EBV-directed cellular immunity compared to healthy controls (34, 35). This finding is consistent with the studies on the association between SLE and cancer, as EBV has been verified in the process of some malignancies, such as Burkitt's lymphoma, Hodgkin’s disease, and nasopharyngeal and gastric carcinomas (36). Our patient was positive for EBV-IgM at the time of the initial diagnosis of lymphoma. No agreement has yet been reached regarding the relationship between SLE disease activity, immunosuppressive intensity, and cancer incidence (1), although higher SLE activity and the use of cyclophosphamide may promote tumor progression (37, 38). Moreover, secondary Sjögren's syndrome may increase the risk of lymphoma because of B-cell clonal hyperplasia (37), which may also explain why lymphoma in patients may be easier to combine with or develop into Sjögren's syndrome during the process of treatment. Our patient was also considered to have developed secondary Sjögren's syndrome.

In conclusion, the present case further supports the existence of an inextricable link between autoimmune diseases and hematological malignancies as changes in the hematopoietic microenvironment may lead to immune system disorders. Despite the low frequency of SLE in children with lymphoma, this process may be induced through a common mechanism, including B-cell cloning and proliferation. Therefore, hematologists and rheumatologists should be aware of the occurrence of both cancer and SLE during patient follow-up.

## Data Availability

The original contributions presented in the study are included in the article/Supplementary Material, and further inquiries can be directed to the corresponding author.
